# Development and validation of a nomogram for overall survival risk stratification in patients with brain metastases undergoing radiotherapy

**DOI:** 10.3389/fmed.2026.1834582

**Published:** 2026-07-23

**Authors:** Mengdie Zhao, Yanqun Zhang, Xiaoyu Zhong, Xianwen Zhang, Shun Li, Ziwen Guo, Zhifei Huang, Miao Li

**Affiliations:** 1 The First Affiliated Hospital of Bengbu Medical University, Bengbu, Anhui, China; 2 The Second Affiliated Hospital of Wannan Medical College, Wuhu, Anhui, China

**Keywords:** brain metastases, LASSO, nomogram, overall survival, radiotherapy, random survival forest

## Abstract

**Background:**

Radiotherapy is a primary treatment modality for patients with brain metastases (BMs). However, individualized prediction of overall survival (OS) in this patient cohort remains a considerable clinical challenge. This study aimed to develop a prognostic model for estimating OS in patients with BMs receiving radiotherapy.

**Methods:**

A retrospective cohort study was conducted, enrolling 298 patients with radiologically confirmed BMs who received radiotherapy from January 2020 to December 2021. Least absolute shrinkage and selection operator (LASSO) Cox regression was used to identify key prognostic factors for OS. A nomogram was developed based on multivariate Cox regression models to predict 1-, 2-, and 3-year OS. The nomogram’s performance was assessed using time-dependent receiver operating characteristic (ROC) curves, calibration plots, and decision curve analysis (DCA). A random survival forest (RSF) model was additionally used to evaluate the relative importance of each prognostic predictor.

**Results:**

LASSO regression identified six key prognostic variables: sex, primary tumor type, graded prognostic assessment (GPA) class, targeted/immunotherapy administration, primary tumor control status, and post-radiotherapy systemic therapy. All six variables were independently associated with OS. The nomogram showed moderate discriminative ability, with area under the curve (AUC) values of 0.773, 0.763, and 0.723 for 1-, 2-, and 3-year OS, respectively. Calibration plots showed reasonable agreement between predicted and observed OS outcomes. DCA suggested potential clinical net benefit across the assessed threshold probabilities. Using a total score cutoff of 224, patients were stratified into high-risk and low-risk groups, with significantly different OS between the two groups (*P* < 0.001). RSF analysis indicated that sex and primary tumor type were the most informative predictors of OS.

**Conclusion:**

The developed prognostic nomogram integrates readily available clinical and treatment-related characteristics to estimate OS in radiotherapy-treated patients with BMs. This model may assist individualized risk stratification and clinical decision-making, although further external validation is needed. An interactive dynamic nomogram is accessible online at: https://mengdie710.shinyapps.io/DynNomapp/

## 1 Introduction

Brain metastases (BMs) occur in around one-fifth of individuals diagnosed with cancer, making them the most frequent type of intracranial tumor seen in adults (1). These secondary tumors typically originate from primary cancers such as those of the lung, breast, and colon, as well as from melanoma and renal cell carcinoma (1). In the United States alone, brain metastases are identified in roughly 8–10% of all cancer patients each year, leading to about 200,000 new diagnoses annually (2). For example, over a quarter of patients with metastatic melanoma or lung adenocarcinoma show evidence of brain involvement, whereas the figure is closer to 10% for renal cell carcinoma and about 7% for breast cancer (3, 4, 5). Treating brain metastases poses significant difficulties, largely because of the high degree of variability within the patient group. These lesions can originate from a diverse range of primary tumors, and many patients have undergone multiple prior systemic therapies, often resulting in treatment resistance across several lines of therapy. Given that intracranial progression contributes substantially to mortality in patients with BMs, this condition remains a major clinical challenge in contemporary oncology (6). Although treatment options have expanded to include surgery, radiotherapy, targeted therapy, and immunotherapy, survival remains highly variable and is still unfavorable in many patients with BMs (1).

While certain targeted treatments have shown efficacy within the brain itself, numerous standard chemotherapy drugs continue to face challenges in effectively crossing the blood-brain barrier (7). Consequently, interventions focused on the local site, including radiation therapy and surgical procedures, continue to play a crucial role in the management of brain metastases, even though these approaches can potentially impact a patient’s overall well-being (8–10). Treatment selection is closely linked to expected survival: patients with very limited survival may derive little benefit from intensive intracranial treatment, whereas those with longer expected survival may be considered for more active local and systemic strategies (11, 12). Current guidelines also emphasize individualized decision-making based on neurological symptoms, intracranial disease burden, extracranial disease status, performance status, and available systemic treatment options. Therefore, reliable survival estimation is essential for balancing treatment intensity, expected benefit, and patient burden.

Survival after BMs is shaped by both baseline disease characteristics and subsequent treatment exposure. Existing prognostic frameworks, such as the Recursive Partitioning Analysis (RPA) and the Graded Prognostic Assessment (GPA), integrate variables including patient age, functional status, presence of systemic disease, and the extent of intracranial disease burden (13, 14). More recent diagnosis-specific GPA models further recognize the prognostic role of primary tumor type and tumor-specific molecular features (15). However, these systems mainly provide group-level risk stratification, and their use in current practice may be limited when systemic disease control and post-radiotherapy treatment are important determinants of outcome.

Nomograms have also been developed to provide more individualized survival estimates for patients with BMs. Barnholtz-Sloan et al. established an individualized survival nomogram using RTOG trial data (16), and subsequent studies extended this approach to patients with non-surgical NSCLC receiving radiotherapy or colorectal cancer brain metastases (17, 18). Other models were developed for patients treated with WBRT or SRS, or incorporated radiomic, molecular, and volumetric variables to refine prediction (19–21). While these efforts have expanded the scope of tailored prognosis evaluation, the variables used and the specific patient groups targeted often differ and are not universally applicable. In everyday clinical radiotherapy settings, a predictive model that relies on routinely accessible clinical and treatment-related data may thus continue to hold practical value.

This research aimed to create and perform an internal validation of a nomogram designed to forecast overall survival (OS) within a real-world patient population diagnosed with brain metastases (BMs) and undergoing radiotherapy. The model focused on routinely available variables, including GPA class, primary tumor type, primary tumor control, targeted/immunotherapy, and post-radiotherapy systemic therapy, and was further explored using random survival forest analysis to assess the relative contribution of each predictor.

## 2 Materials and methods

### 2.1 Study design and Participants

This retrospective cohort study included patients diagnosed with brain metastases who received radiotherapy at the Department of Radiation Oncology, The First Affiliated Hospital of Bengbu Medical University, from January 2020 to December 2021. A total of 298 patients met the eligibility criteria and were included in the final analysis. The study was approved by the Ethics Committee of The First Affiliated Hospital of Bengbu Medical University (Ethical Approval No. 2022–258). Written informed consent for this retrospective analysis was waived by the ethics committee because only de-identified clinical data were used. The study was conducted in accordance with the Declaration of Helsinki and institutional requirements. Because this was a retrospective cohort study, no formal a priori sample size calculation was performed. Instead, all consecutive eligible patients treated during the study period were included. The adequacy of the sample size for model development was assessed according to the number of observed death events relative to the number of predictors retained in the final Cox model (22–24).

**FIGURE 1:**
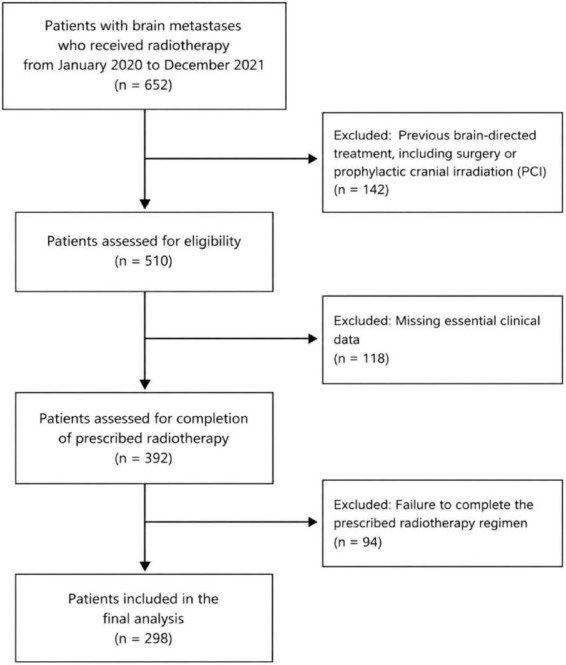
Patient selection flowchart. A total of 652 patients with brain metastases who received radiotherapy between January 2020 and December 2021 were initially identified. After exclusion of patients with previous brain-directed treatment (*n* = 142), missing essential clinical data (*n* = 118), and failure to complete the prescribed radiotherapy regimen (*n* = 94), 298 patients were included in the final analysis.

Inclusion criteria were as follows: (1) pathologically or cytologically confirmed primary malignancy; (2) diagnosis of brain metastases based on imaging findings from contrast-enhanced computed tomography (CT) or magnetic resonance imaging (MRI); (3) age ≥18 years at the initiation of radiotherapy; (4) Karnofsky Performance Status (KPS) score ≥ 60, or ≥ 40 when the reduced performance status was mainly attributable to intracranial tumor burden and the patient was considered able to tolerate radiotherapy; (5) ability to tolerate radiotherapy simulation and immobilization procedures; (6) availability of complete clinical and treatment-related data. Exclusion criteria included: (1) previous brain-directed treatments, including surgery or prophylactic cranial irradiation (PCI); (2) missing essential clinical data; (3) failure to complete the prescribed radiotherapy regimen. Patients who discontinued radiotherapy were not included because their planned treatment exposure and post-radiotherapy status could not be consistently assessed (Figure 1).

### 2.2 Radiotherapy protocol

All patients received radiotherapy for brain metastases using standardized institutional procedures. Simulation was performed using a large-bore CT simulator (Toshiba, Japan), with patients positioned supine and immobilized using a thermoplastic mask to ensure reproducibility. The planning CT images (slice thickness ≤ 3 mm, from the vertex to the lower margin of C2) were transferred to the Pinnacleł treatment planning system (Philips, Netherlands) for target delineation and dose calculation.

Target volumes were defined according to clinical treatment strategies. For WBRT, the clinical target volume (CTV) encompassed the entire brain parenchyma, and the planning target volume (PTV) was generated by expanding the CTV by 3–5 mm. For focal radiotherapy, the gross tumor volume (GTV) corresponded to metastatic lesions identified on MRI or contrast-enhanced CT, with a similar 3–5 mm margin to form the PTV. In patients receiving both WBRT and local boost (WBRT + Boost), separate PTVs were defined for whole-brain and focal lesions (PTV1 and PTV2, respectively). Dose constraints for organs at risk (OARs) adhered to institutional and international guidelines (e.g., brainstem ≤ 45 Gy, optic structures ≤ 50 Gy, lenses ≤ 6 Gy).

Treatment was delivered using either a Siemens Artiste or Elekta Precise linear accelerator, employing techniques such as two-dimensional radiotherapy (2D-RT), three-dimensional conformal radiotherapy (3D-CRT), intensity-modulated radiotherapy (IMRT), or stereotactic radiotherapy (SRT), depending on lesion characteristics. The total dose and fractionation schemes were individualized based on tumor burden, lesion location, primary tumor type, and patient performance status. All doses were converted into equivalent doses in 2 Gy fractions (EQD2) using a linear-quadratic model (α/β = 10 for tumor tissue, 2 for normal brain).

Image-guided radiotherapy (IGRT) using cone-beam CT was performed before each fraction to ensure accurate patient positioning. All treatment plans were reviewed by radiation oncologists and medical physicists to confirm target coverage and adherence to quality assurance protocols. The choice of WBRT, local radiotherapy, or WBRT combined with local boost was made according to intracranial disease burden, lesion size and location, performance status, extracranial disease status, and expected survival (25). Treatment decisions were generally consistent with contemporary recommendations for the management of brain metastases, while final radiotherapy plans were individualized according to institutional practice and multidisciplinary assessment (12).

### 2.3 Data collection

Baseline clinical data were extracted from the hospital’s electronic medical record system. Demographic information included age and sex. Age was grouped as <60 and ≥60 years, a cutoff commonly used in brain metastasis prognostic assessment and consistent with the age distribution of this cohort. Body mass index (BMI) was calculated as weight in kilograms divided by height in meters squared and grouped according to the World Health Organization classification: underweight (<18.5 kg/m^2^), normal weight (18.5–24.9 kg/m^2^), and overweight/obese (≥25 kg/m^2^) (26).

Lifestyle factors such as smoking status and alcohol consumption (yes or no) were recorded based on patient self-report at admission. Comorbidities, including hypertension, diabetes mellitus, and cardiovascular disease, were determined from documented medical history or active treatment at the time of radiotherapy initiation.

Tumor-related characteristics were evaluated using contrast-enhanced brain magnetic resonance imaging (MRI) or computed tomography (CT) scans. The primary tumor type was categorized as lung, breast, or other solid tumors. Less common primary tumors were grouped into the “other solid tumor” category because of the limited number of cases and the retrospective structure of the dataset. The maximum diameter of brain metastases was defined as the largest measurable lesion on axial MRI or CT images and was grouped as <20 mm and ≥20 mm to provide a clinically interpretable indicator of intracranial tumor burden. The number of brain metastases was classified as single or multiple based on radiological assessment. Extracranial metastases were identified through systemic imaging (e.g. chest/abdomen CT or PET-CT). The status of primary tumor control was classified into three categories: controlled (stable or responding disease), uncontrolled (progressive disease), and newly diagnosed (primary cancer identified concurrently with brain metastasis), as determined by imaging and oncologist documentation.

Functional and prognostic status was assessed using three validated systems. KPS was assessed by radiation oncologists at the initiation of radiotherapy. KPS was grouped as ≤ 70 and > 70 for prognostic analysis, reflecting impaired versus relatively preserved functional status in established brain metastasis prognostic indices. KPS evaluates a patient’s ability to perform ordinary tasks and ranges from 0 (death) to 100 (normal functioning) (27).

RPA class was assigned according to the Radiation Therapy Oncology Group (RTOG) classification, incorporating age, KPS, status of extracranial metastases, and control of the primary tumor (13). GPA scores were calculated using tumor-specific GPA indices, integrating age, KPS, number of brain metastases, and presence of extracranial disease. For clinical interpretability and to avoid sparse subgroups, GPA scores were grouped as 0–1.0, 1.5–2.5, and 3.0–4.0, with higher scores indicating a more favorable prognosis (14).

Treatment-related variables included radiotherapy modality (whole-brain radiotherapy [WBRT], local radiotherapy, or WBRT combined with local boost), concurrent chemotherapy, targeted/immunotherapy, and receipt of systemic therapy following radiotherapy.

### 2.4 Follow-up and outcome

Patients were followed up through telephone interviews, electronic medical record reviews, and routine outpatient visits. The last date of follow-up was April 30, 2025. The primary outcome was overall survival (OS), defined as the time from the initiation of radiotherapy for brain metastases to death from any cause. Patients who remained alive at the last confirmed follow-up were censored at that date. The number and proportion of censored patients were also reported. All outcomes were independently verified by at least two investigators to ensure accuracy.

### 2.5 Statistical analysis

All statistical evaluations were carried out with the R software platform (version 4.3.1). Categorical data were presented as counts and their corresponding percentages. For every variable, the rate of missing information was below 5%. To address missing values, the multiple imputation technique using chained equations was applied, facilitated by the “mice” package. Every statistical test conducted was two-tailed, and results with a *P*-value lower than 0.05 were deemed to have statistical significance.

To pinpoint the most significant prognostic factors influencing overall survival (OS), a least absolute shrinkage and selection operator (LASSO) Cox regression analysis was performed with the assistance of the “glmnet” package. The selection of the optimal penalty parameter (λ) was achieved through a five-fold cross-validation process, adhering to the one-standard-error (1-SE) criterion for the final determination. The model incorporated a comprehensive set of variables, such as patient demographics, existing medical conditions, the type of radiotherapy administered, the specific nature of the primary tumor, the presence of metastases outside the brain, Karnofsky Performance Status (KPS), Recursive Partitioning Analysis (RPA) classification, Graded Prognostic Assessment (GPA) classification, the use of concurrent chemotherapy, the application of targeted or immunotherapy, the largest tumor dimension, the total count of brain metastases, the status of primary tumor control, and systemic treatments received following radiotherapy.

Variables identified through the LASSO procedure were integrated into both univariate and multivariate Cox proportional hazards regression models to compute hazard ratios (HRs) along with their corresponding 95% confidence intervals (CIs). Differences in overall survival (OS) across various strata of the variables were evaluated using Kaplan–Meier curves and log-rank tests, which were executed with the assistance of the “survival” and “survminer” software packages. To evaluate the robustness of the final Cox regression model, the events per variable (EPV) metric was determined based on the count of recorded deaths and the number of predictor variables included in the final multivariable model. The stability of the model was also examined in the context of earlier methodological studies, which indicate that the necessary sample size for constructing a prediction model is influenced not merely by the events per variable but also by the overall complexity of the model and the anticipated degree of overfitting (22–24). The assumption of proportional hazards for the final Cox model was assessed through the examination of Schoenfeld residuals.

A predictive nomogram was constructed employing the multivariate Cox regression model via the “rms” package, designed to estimate the probabilities of overall survival (OS) at one, two, and three years. Utilizing the “DynNom” package, an interactive and dynamic iteration of this nomogram was created. For the purpose of personalized risk categorization, a cumulative score was computed for every individual patient. The “maxstat” package was then applied to identify the most effective threshold, thereby dividing the patient cohort into distinct low-risk and high-risk categories.

The nomogram’s predictive accuracy was assessed through time-dependent receiver operating characteristic (ROC) curves, produced by the “timeROC” package, which provided area under the curve (AUC) metrics for the one-, two-, and three-year time points. Calibration plots, generated with the “rms” package, were used to examine the concordance between the model’s predicted survival outcomes and the actual observed survival data. To appraise the clinical applicability of the nomogram, decision curve analysis (DCA) was performed using the “ggDCA” package, calculating the net benefit over a spectrum of probability thresholds.

Finally, a prognostic model based on random survival forest (RSF) was developed with the “randomForestSRC” package to evaluate the relative significance of each predictive variable. The importance of variables (VIMP) and their minimal depth metrics were employed to order the predictors based on their impact on forecasting survival outcomes.

## 3 Results

### 3.1 Baseline characteristics

A total of 298 patients with brain metastases who received radiotherapy were included in the analysis (Table 1). The mean age was 60.29 ± 9.99 years, with a range of 26–80 years; 52.7% of patients were aged 60 years or older. Male patients accounted for 53.7% of the cohort. Lung cancer was the most common primary tumor type (77.2%), followed by breast cancer (12.8%). At baseline, 75.2% of patients had a Karnofsky Performance Status (KPS) score > 70. According to the RPA, 65.8% were classified as class II. Based on the GPA, 59.7% of patients were categorized as intermediate risk (GPA 1.5–2.5). Additionally, 68.5% had multiple brain metastases, and 69.5% had controlled primary tumors at the time of radiotherapy. Regarding treatment, half of the patients (50.0%) received targeted therapy or immunotherapy, and the majority (83.9%) received systemic therapy following radiotherapy. The last follow-up date was April 30, 2025. During follow-up, 242 patients died and 56 patients were censored, corresponding to a censoring rate of 18.8%. The censored patients were those who remained alive at their last confirmed follow-up.

**TABLE 1:** Baseline characteristics of patients with brain metastases receiving radiotherapy.

Characteristics	N (%)
Age group	
< 60 years	141 (47.3%)
≥ 60 years	157 (52.7%)
Sex	
Female	138 (46.3%)
Male	160 (53.7%)
BMI category	
< 18.5 kg/m^2^	18 (6.0%)
18.5–24.9 kg/m^2^	177 (59.4%)
≥ 25 kg/m^2^	103 (34.6%)
Smoking status	
No	277 (93.0%)
Yes	21 (7.0%)
Alcohol use	
No	285 (95.6%)
Yes	13 (4.4%)
Hypertension	
No	234 (78.5%)
Yes	64 (21.5%)
Diabetes mellitus	
No	272 (91.3%)
Yes	26 (8.7%)
Cardiovascular disease	
No	287 (96.3%)
Yes	11 (3.7%)
Radiotherapy type	
Whole brain	110 (36.9%)
Local	97 (32.6%)
WBRT + local	91 (30.5%)
Primary tumor type	
Lung	230 (77.2%)
Breast	38 (12.8%)
Other	30 (10.1%)
Other metastases	
No	144 (48.3%)
Yes	154 (51.7%)
KPS class	
≤ 70	74 (24.8%)
> 70	224 (75.2%)
RPA class	
I	48 (16.1%)
II	196 (65.8%)
III	54 (18.1%)
GPA class	
0–1	70 (23.5%)
1.5–2.5	178 (59.7%)
3–4	50 (16.8%)
Concurrent chemotherapy	
No	248 (83.2%)
Yes	50 (16.8%)
Targeted/Immunotherapy	
No	149 (50.0%)
Yes	149 (50.0%)
Max tumor diameter	
< 20 mm	121 (40.6%)
≥ 20 mm	177 (59.4%)
Number of brain metastases	
Single	94 (31.5%)
Multiple	204 (68.5%)
Primary tumor control	
Controlled	207 (69.5%)
Uncontrolled	44 (14.8%)
Newly diagnosed	47 (15.8%)
Post-RT systemic therapy	
No	48 (16.1%)
Yes	250 (83.9%)

BMI, body mass index; KPS, Karnofsky Performance Status; RPA, Recursive Partitioning Analysis; GPA, Graded Prognostic Assessment; RT, radiotherapy; WBRT, whole-brain radiotherapy.

### 3.2 Variable selection using LASSO regressions

**FIGURE 2:**
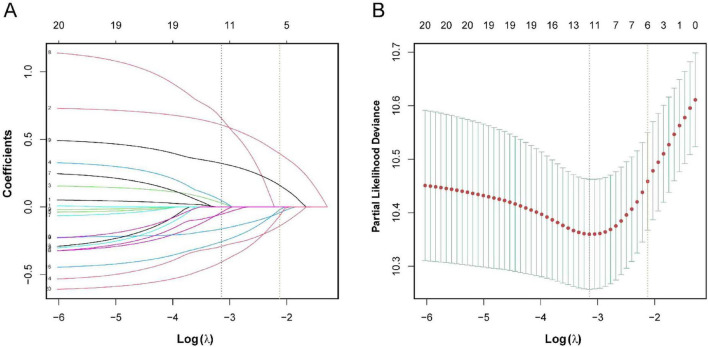
Selection of prognostic variables using LASSO regression. LASSO regression with five-fold cross-validation was used to identify prognostic variables for overall survival. **(A)** LASSO coefficient profiles of the 20 candidate variables plotted against the log-transformed λ sequence. **(B)** Mean cross-validated partial likelihood deviance plotted against log(λ), with vertical dashed lines indicating the values of λ that correspond to the minimum deviance and the most regularized model within one standard error of the minimum (λ = 0.120, 1-SE criterion). Six non-zero coefficients were retained at the 1-SE criterion.

To identify the most informative prognostic variables for overall survival, the LASSO-Cox regression model was applied with five-fold cross-validation (Figure 2). Based on the one-standard-error (1-SE) criterion, the optimal penalization parameter was λ = 0.120, which yielded a more parsimonious model comprising six non-zero coefficients. The selected predictors included sex, primary tumor type, GPA class, targeted/immunotherapy, primary tumor control status, and post-radiotherapy systemic therapy. Radiotherapy modality, maximum tumor diameter, and number of brain metastases were also evaluated during variable selection but were not retained at the 1-SE criterion. The six selected variables were subsequently entered into the multivariable Cox proportional hazards model and nomogram construction.

### 3.3 Kaplan–Meier survival analysis and cox regression

**FIGURE 3:**
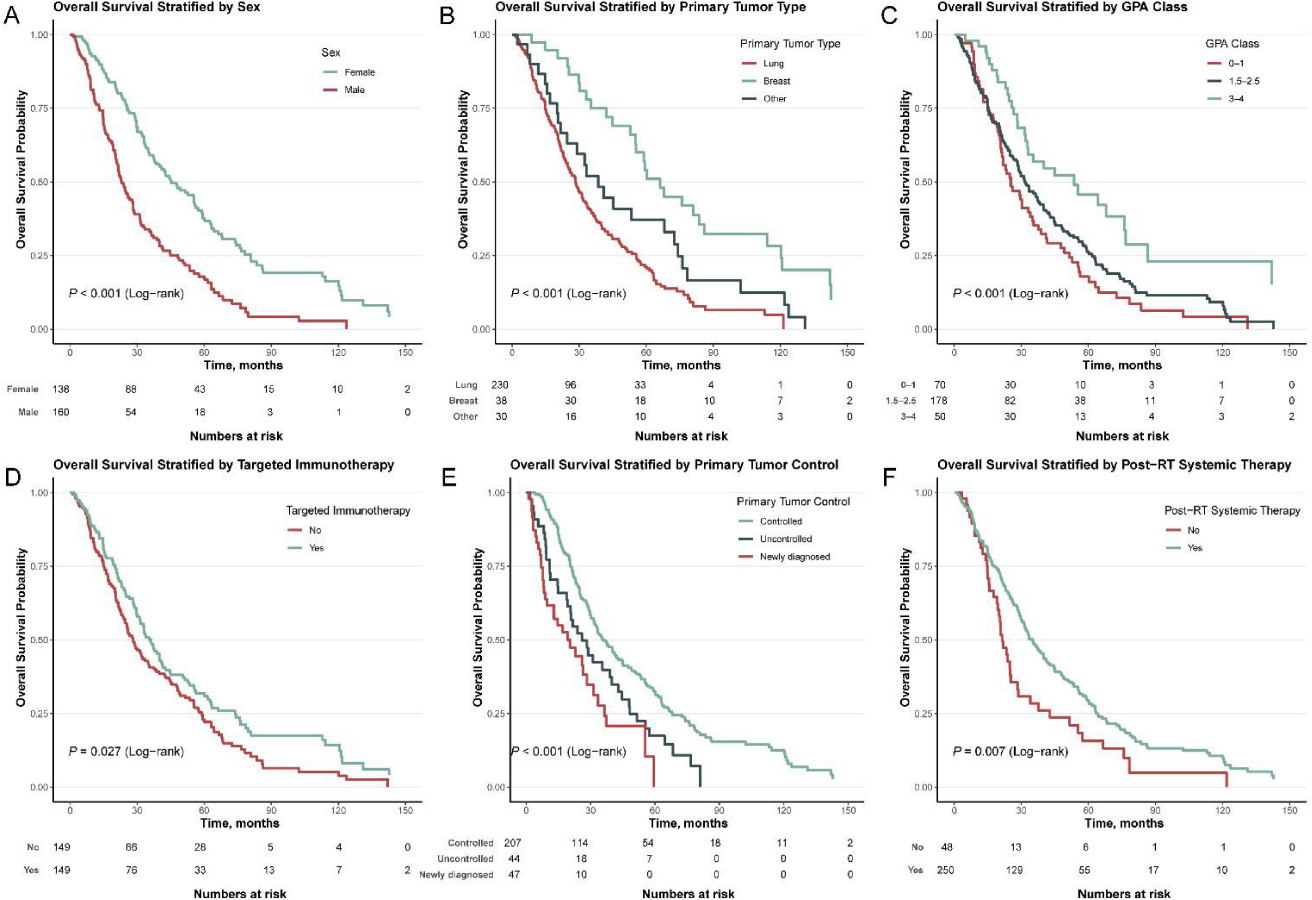
Kaplan–Meier survival curves based on LASSO-selected variables. Kaplan–Meier curves for overall survival stratified by **(A)** sex, **(B)** primary tumor type, **(C)** GPA class, **(D)** targeted/immunotherapy, **(E)** primary tumor control status, and **(F)** post-radiotherapy systemic therapy. All variables showed significant survival differences (log-rank *P* < 0.05).

Kaplan–Meier analysis showed that OS differed significantly across the six variables identified by LASSO regression (Figure 3). Male sex, lung cancer, and newly diagnosed or uncontrolled primary tumors were associated with shorter OS (all *P* < 0.001). Conversely, higher GPA class, receipt of targeted/immunotherapy (*P* = 0.027), and post-radiotherapy systemic therapy (*P* = 0.007) were associated with longer survival. Univariate Cox regression analyses identified all six variables as significantly associated with OS (Table 2). During follow-up, 242 deaths were recorded. Based on 242 observed death events and six predictors in the final Cox model, the EPV was 40.3. The event-to-parameter ratio remained above 10 even after accounting for categorical predictors. In the multivariate model, male sex (HR = 1.82; 95% CI, 1.36–2.42) and newly diagnosed primary tumors (HR = 2.54; 95% CI, 1.70–3.79) were independently associated with poorer OS. By contrast, breast cancer (HR = 0.549; 95% CI, 0.34–0.90), other primary tumor types (HR = 0.62; 95% CI, 0.39–0.97), GPA class 3–4 (HR = 0.42; 95% CI, 0.27–0.66), receipt of targeted/immunotherapy (HR = 0.65; 95% CI, 0.50–0.85), and receipt of post-radiotherapy systemic therapy (HR = 0.58; 95% CI, 0.41–0.82) were independently associated with improved OS. The proportional hazards assumption was examined using Schoenfeld residuals. The global test was not statistically significant (*P* = 0.355), and no clear evidence of non-proportionality was observed for post-radiotherapy systemic therapy (*P* = 0.549).

**TABLE 2:** Univariate and multivariate cox proportional hazards regression analyses for overall survival among patients with brain metastases receiving radiotherapy.

V Category	Univariate analysis		Multivariate analysis	
	HR (95%CI)	*P*-value	HR (95%CI)	*P*-value
Sex, Male Primary tumor type	2.10 (1.62–2.74)	< 0.001	1.82 (1.36–2.42)	< 0.001
		< 0.001		0.013
Lung	1.00 (Reference)		1.00 (Reference)	
Breast	0.35 (0.23–0.54)	< 0.001	0.549 (0.34–0.90)	0.016
Other	0.69 (0.45–1.04)	0.075	0.62 (0.39–0.97)	0.035
GPA class 0–1	1.00 (Reference)	0.001	1.00 (Reference)	< 0.001
	
1.5–2.5	0.81 (0.60–1.09)	0.157	0.79 (0.58–1.06)	0.115
3–4	0.44 (0.28–0.68)	< 0.001	0.42 (0.27–0.66)	< 0.001
Targeted/immunotherapy	0.75 (0.58–0.97)	0.027	0.65 (0.50–0.85)	0.002
Primary tumor control Controlled	1.00 (Reference)	< 0.001	1.00 (Reference)	<0.001
	
Uncontrolled	1.61 (1.13–2.29)	0.008	1.14 (0.78–1.64)	0.504
Newly diagnosed	2.35 (1.60–3.44)	< 0.001	2.54 (1.70–3.79)	< 0.001
Post-RT systemic therapy	0.64 (0.45–0.89)	0.008	0.58 (0.41–0.82)	0.002

Hazard ratios (HRs) and 95% confidence intervals (CIs) were derived from Cox proportional hazards models. Variables retained by LASSO were included in the multivariate Cox proportional hazards model. The reference categories for categorical variables are indicated. HR, hazard ratio; CI, confidence interval; RPA, Recursive Partitioning Analysis; GPA, Graded Prognostic Assessment; RT, radiotherapy.

### 3.4 Nomogram for predicting overall survival

**FIGURE 4:**
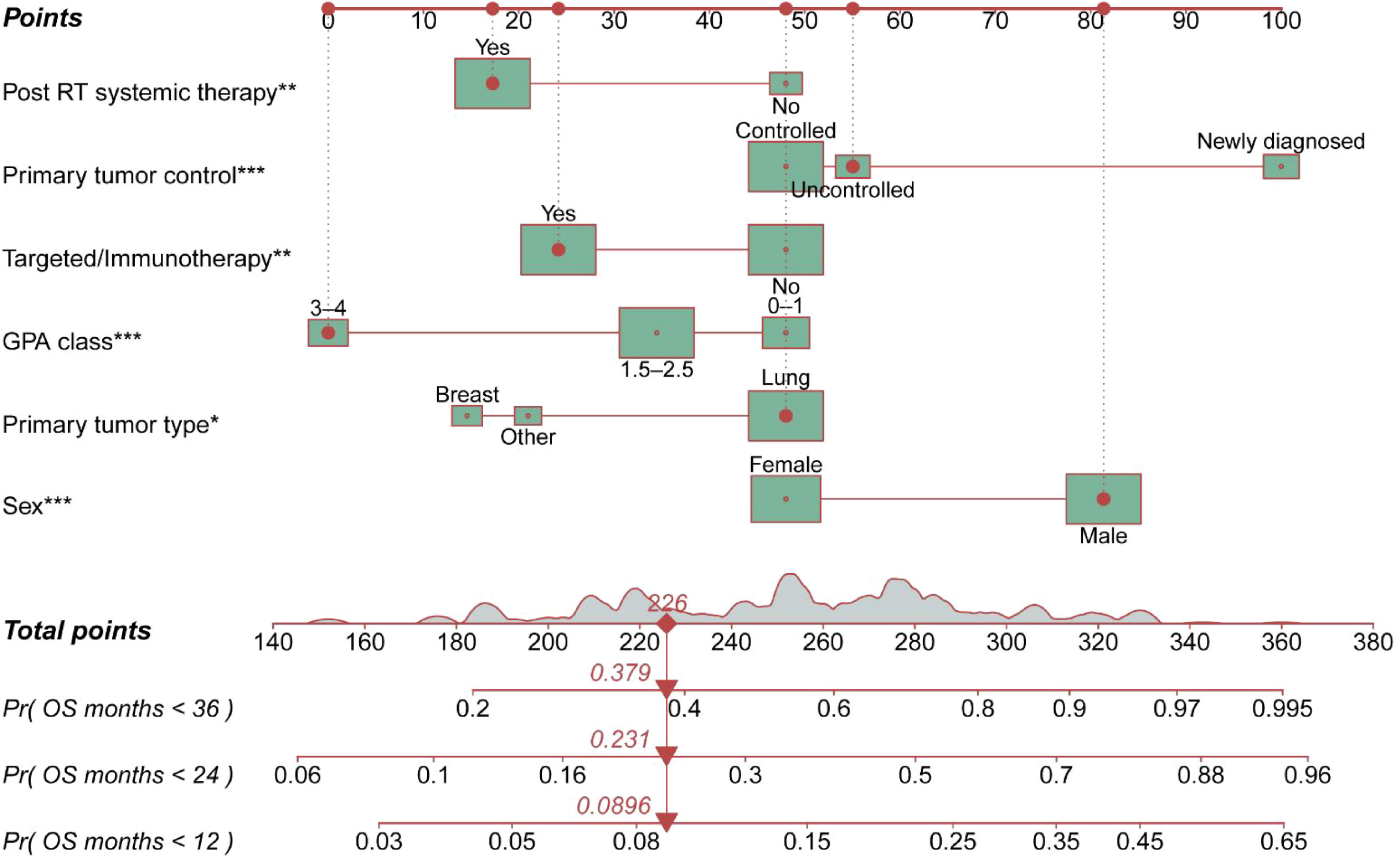
Nomogram for predicting 1-, 2-, and 3-year overall survival. A prognostic nomogram was developed based on multivariate Cox analysis to estimate the probability of 1-, 2-, and 3-year overall survival among patients with brain metastases. Each variable corresponds to a specific number of points on the top scale. Total points are summed to estimate survival probability. For illustration, a hypothetical patient scoring 226 points has estimated 1-, 2-, and 3-year survival probabilities of 37.9, 23.1, and 8.96%, respectively. An interactive dynamic version is available at: https://mengdie710.shinyapps.io/DynNomapp/. * *P* < 0.05; **P < 0.01; ***P < 0.001.

A nomogram was constructed incorporating the six independent prognostic factors identified in the multivariate Cox analysis to estimate 1-, 2-, and 3-year overall survival probabilities (Figure 4). Each variable contributes a specific number of points, and the total score corresponds to predicted survival probabilities. For example, a male patient with lung cancer (primary tumor), GPA score of 1.5–2.5, uncontrolled primary tumor, no targeted/immunotherapy, and no post-radiotherapy systemic therapy would receive a total of 226 points. Based on the nomogram, the estimated 1-, 2-, and 3-year survival probabilities for this patient are approximately 37.9, 23.1 and 8.96%, respectively.

To facilitate individualized prognostic evaluation in clinical practice, we also developed an interactive web-based dynamic nomogram available at: https://mengdie710.shinyapps.io/DynNomapp/.

### 3.5 Risk stratification based on nomogram-derived scores

**FIGURE 5:**
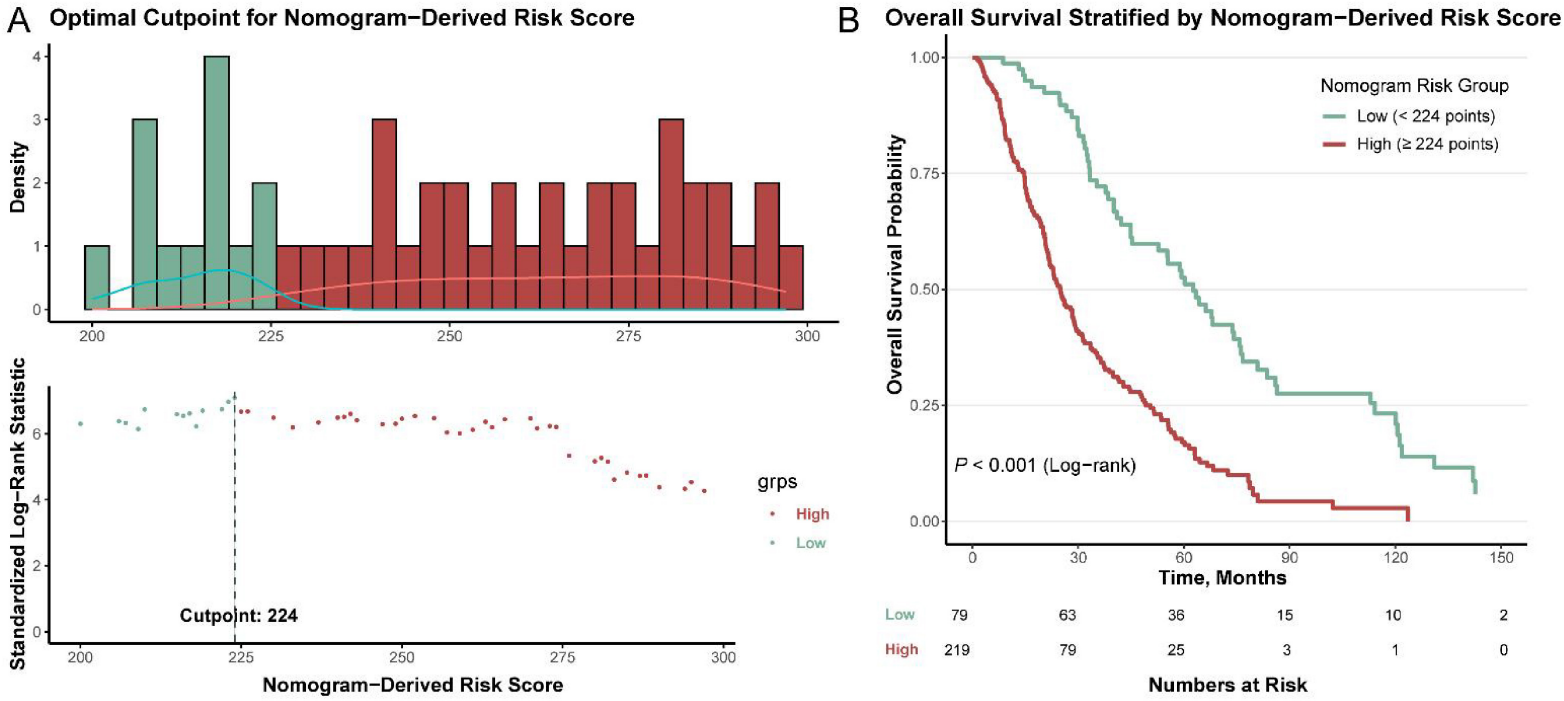
Risk stratification based on nomogram-derived scores. **(A)** Distribution of total nomogram-derived risk scores and optimal cutoff value (224 points) determined by maximally selected rank statistics. **(B)** Kaplan–Meier curves show significant survival differences between low-risk ( < 224) and high-risk ( ≥ 224) groups (log-rank *P* < 0.001).

To facilitate clinical risk stratification, total risk scores were calculated for each patient based on the established nomogram. An optimal cutoff value of 224 points was identified using maximally selected rank statistics, dividing patients into low-risk (<224 points) and high-risk (≥224 points) groups (Figure 5A). Kaplan–Meier survival analysis revealed a significant difference in overall survival between the two groups, with high-risk patients demonstrating markedly worse outcomes (*P* < 0.001; Figure 5B). These results indicate that the nomogram-derived score could separate patients with distinct survival outcomes after radiotherapy.

### 3.6 Evaluation of predictive performance of the nomogram

**FIGURE 6:**
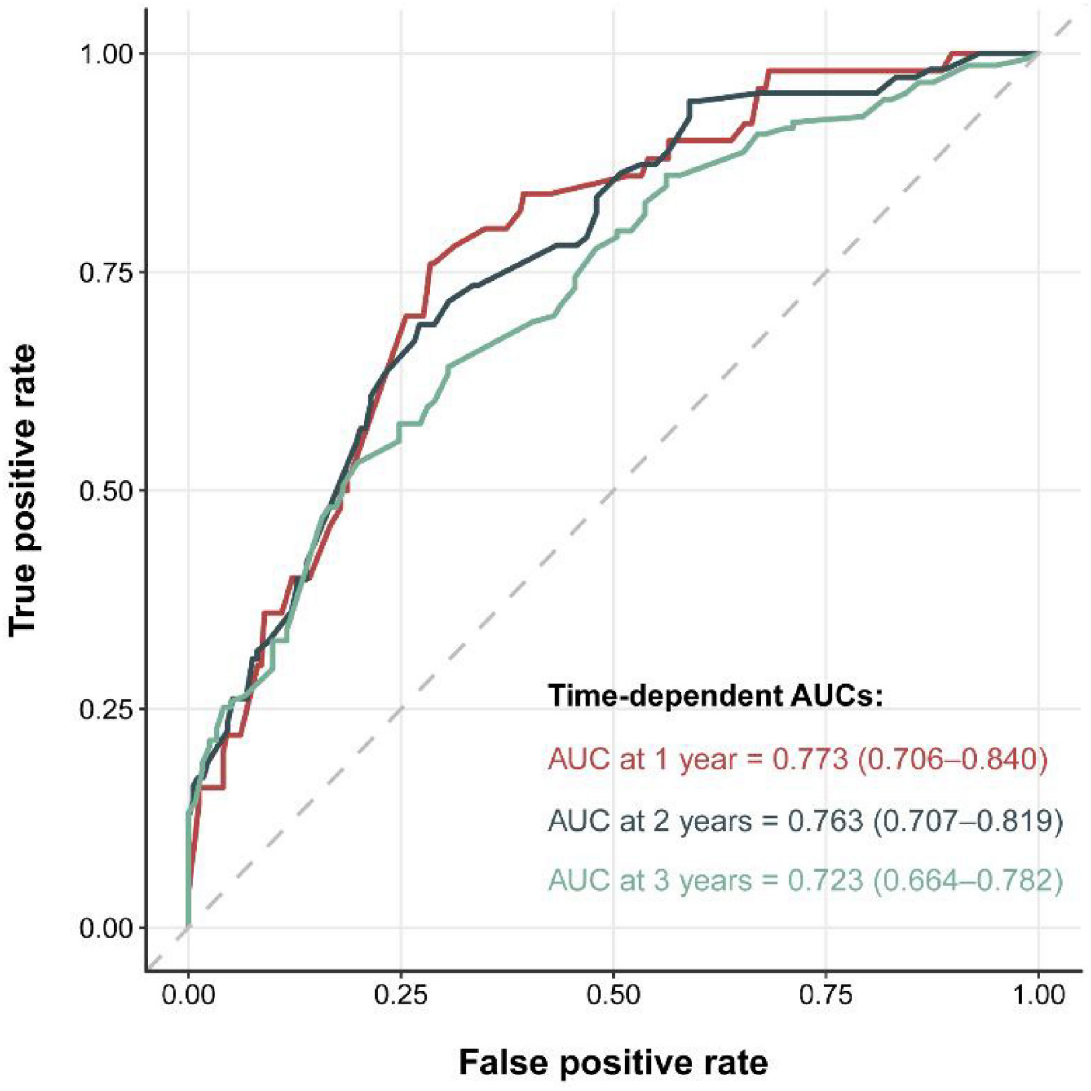
Time-dependent ROC curves for nomogram performance. Time-dependent receiver operating characteristic (ROC) curves for 1-, 2-, and 3-year overall survival prediction. The nomogram showed moderate discriminative ability with AUCs of 0.773, 0.763, and 0.723, respectively.

**FIGURE 7:**
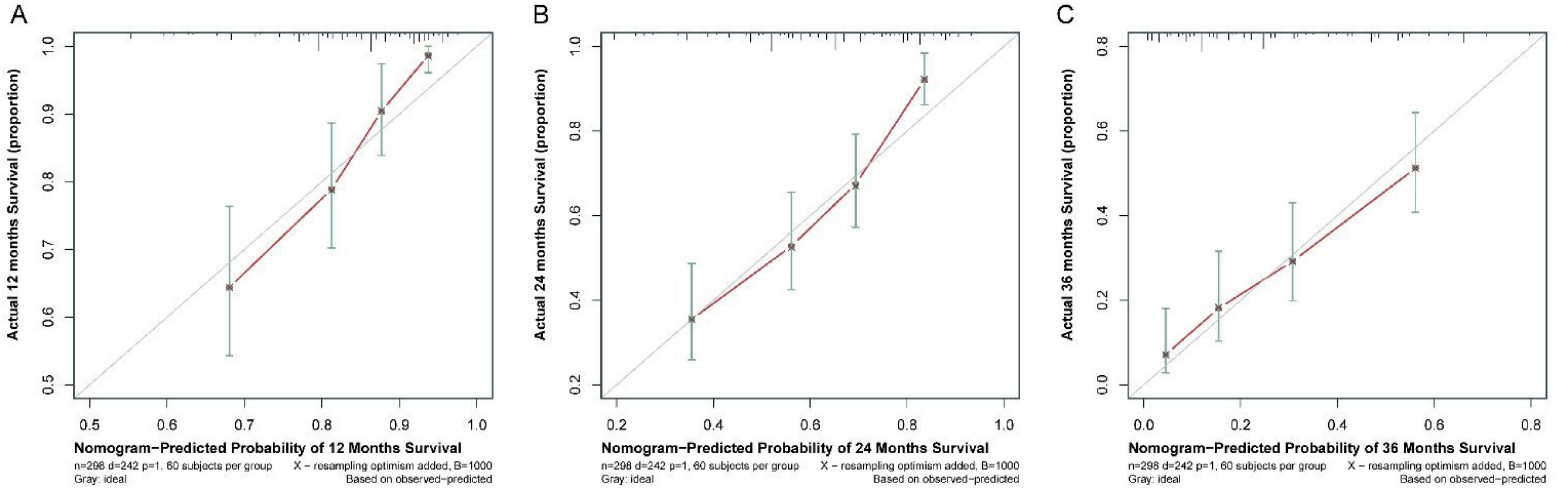
Calibration curves of the nomogram. Calibration plots comparing predicted versus observed overall survival probabilities at 1, 2, and 3 years. The 45-degree diagonal line represents perfect prediction, and the calibration curves showed reasonable agreement between predicted and observed probabilities. **(A)** 12-month survival, **(B)** 24-month survival, **(C)** 36-month survival.

**FIGURE 8:**
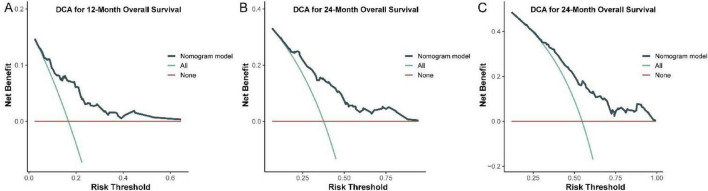
Decision curve analysis for clinical utility of the nomogram. Decision curve analysis (DCA) for 1-, 2-, and 3-year survival prediction. The nomogram showed potential net benefit across a range of threshold probabilities compared with the “treat-all” or “treat-none” strategies. **(A)** 12-month survival, **(B)** 24-month survival (full model), **(C)** 24-month survival (alternative threshold analysis).

The time-dependent ROC analysis showed moderate discriminative ability of the nomogram, with AUC values of 0.773 (95% CI, 0.706–0.840), 0.763 (95% CI, 0.707–0.819), and 0.723 (95% CI, 0.664–0.782) for predicting 1-, 2-, and 3-year overall survival, respectively (Figure 6). Calibration curves showed reasonable agreement between the predicted and observed survival probabilities at 1, 2, and 3 years (Figure 7). Furthermore, DCA suggested that the model may provide clinical net benefit across a range of threshold probabilities compared with treat-all or treat-none strategies (Figure 8).

### 3.7 Variable importance assessment based on RSF

**FIGURE 9:**
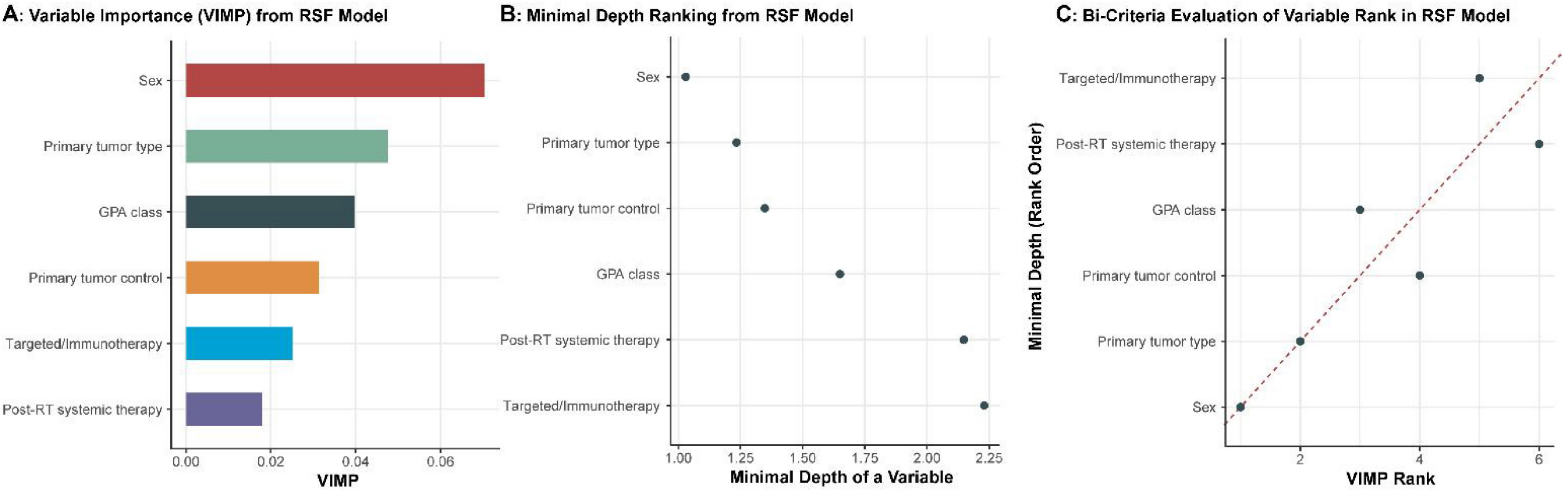
Variable importance based on random survival forest analysis. Random survival forest (RSF) was used to assess the relative importance of the six prognostic variables. **(A)** Variable importance (VIMP) plot showing sex, primary tumor type, and GPA class as the top predictors. **(B)** Minimal depth plot confirming these variables as having the earliest splits in the survival trees. **(C)** Comparative plot showing general agreement between VIMP and minimal depth rankings.

To further evaluate the relative prognostic contribution of each variable, we conducted a RSF analysis. The variable importance plot (Figure 9A) revealed that sex was the most influential predictor, followed by primary tumor type and GPA class. The minimal depth plot (Figure 9B) supported these findings, showing that sex and primary tumor type had the lowest minimal depth values, indicating higher predictive value. Additionally, the comparative plot (Figure 9C) showed general concordance between the VIMP and minimal depth rankings. Taken together, the RSF results suggested that sex and primary tumor type contributed substantially to survival prediction in this radiotherapy-treated cohort.

## 4 Discussion

This research constructed and performed an internal assessment of a prognostic nomogram designed to predict one-, two-, and three-year overall survival for individuals with brain metastases undergoing radiotherapy. The predictive tool integrates six readily accessible clinical and treatment variables: patient gender, the histological type of the primary cancer, the Graded Prognostic Assessment category, the administration of targeted or immunotherapy, the status of primary tumor control, and the use of systemic therapy following radiotherapy. The nomogram demonstrated satisfactory discriminatory ability, achieving area under the curve scores of 0.773, 0.763, and 0.723 for the one-, two-, and three-year time points, respectively.

Calibration plots indicated an acceptable level of concordance between the model’s predictions and the actual observed survival data. Decision curve analysis further implied the model’s possible clinical benefit. Moreover, the risk score generated by the nomogram effectively categorized patients into subgroups with significantly different survival prospects. These results indicate that this model could serve as a useful instrument for stratifying risk in this patient population managed with radiotherapy, pending confirmation through external validation studies.

The evaluation of prognosis in brain metastases has transitioned from collective scoring systems to more personalized predictive tools. Initial frameworks like the RPA and GPA, developed by the RTOG, established critical methods for estimating patient survival based on factors such as functional status, patient age, systemic disease burden, the count of brain lesions, and the nature of the original cancer (13, 14). Although the RPA offered a valuable structure for categorizing risk levels, its somewhat simplistic design constrained the ability to make highly tailored predictions. Later iterations, including the GPA and its specialized versions for specific cancers, integrated further elements like the primary cancer’s histology and molecular biomarkers to enhance the precision of survival forecasts (28). The SIR added considerations for the quantity of brain metastases and the size of the dominant tumor to aid in selecting appropriate candidates for stereotactic radiosurgery (29). While these scoring systems continue to hold clinical relevance, they predominantly capture static, initial clinical and tumor attributes. In modern clinical management, survival outcomes for patients with brain metastases are additionally shaped by control of extracranial disease, accessibility of potent systemic treatments, and therapeutic interventions administered following radiation therapy. Under these circumstances, a predictive model that integrates both initial prognostic indicators and variables associated with the treatment course could yield a more clinically meaningful assessment of survival for individuals with brain metastases receiving radiotherapy.

Published nomograms have provided useful individualized survival estimates for patients with BMs (30). For example, Barnholtz-Sloan et al. developed a nomogram using RTOG trial data to refine prognostic estimation beyond RPA and DS-GPA categories (16). Later studies extended this approach to more specific clinical settings, including non-surgical NSCLC patients receiving radiotherapy, colorectal cancer patients with BMs, and patients treated with WBRT or SRS (17–19). More recently, radiomics-based and molecularly informed nomograms have further expanded the range of candidate predictors, although their application often depends on imaging feature extraction, volumetric assessment, or driver gene information (20, 21). The present model was developed in a real-world cohort of radiotherapy-treated patients and retained variables that are commonly available in routine clinical records, including GPA class, primary tumor control, targeted/immunotherapy, and post-radiotherapy systemic therapy. This structure may be more practical for survival estimation when detailed molecular, radiomic, or volumetric data are incomplete.

Some previous models were designed for endpoints other than OS, such as distant brain failure or the need for salvage WBRT after SRS (31–33). These models are clinically useful for planning intracranial surveillance and salvage treatment, but their endpoints and target populations differ from those of the present study. Our model focused on OS after radiotherapy and therefore retained variables reflecting both systemic disease status and subsequent systemic treatment. This difference may explain why primary tumor control and post-radiotherapy systemic therapy were selected in the final model, whereas radiotherapy modality, maximum tumor diameter, and number of brain metastases were not retained at the 1-SE criterion.

The present model incorporated variables reflecting both disease status and treatment exposure. Primary tumor control is closely related to extracranial disease status and overall prognosis in patients with BMs. Clinical outcomes are also influenced by primary tumor histology, and even variations within the same primary cancer type may affect the likelihood of developing new brain metastases after SRS (34). Sex remained independently associated with OS in the multivariate model and ranked as the most informative predictor in the RSF analysis. Male patients had worse OS than female patients in this cohort. Similar findings have been reported in patients with BMs treated with radiosurgery, where female sex was associated with longer survival (35). In the present cohort, this association may partly reflect differences in primary tumor composition and treatment sensitivity. Lung cancer accounted for most cases, whereas breast cancer represented a smaller but prognostically distinct subgroup. Therefore, the observed sex difference should be interpreted together with tumor type, molecular background, and systemic treatment patterns rather than as an isolated biological effect. The inclusion of targeted/immunotherapy and post-radiotherapy systemic therapy in the final model is clinically plausible. As systemic therapy has improved, survival in patients with BMs is increasingly influenced not only by intracranial disease control but also by extracranial disease burden and the availability of effective systemic agents (9). Immunotherapy has shown therapeutic potential in patients with BMs, and selected targeted agents may also achieve meaningful intracranial activity in molecularly defined tumors (36, 37). The effectiveness of systemic therapy in BMs is still affected by central nervous system drug delivery, blood–brain barrier disruption, and tumor-specific biological features, which may partly explain the variability in treatment response across different primary tumors (38). In a recent study of NSCLC patients with brain metastases, the integration of G-CSF with WBRT and immunotherapy was associated with longer OS, although intracranial response rates were not significantly increased (39). Consistent with this evidence, both targeted/immunotherapy and post-radiotherapy systemic therapy were associated with improved OS in our cohort, supporting the inclusion of treatment-related variables when estimating prognosis in contemporary radiotherapy-treated patients.

The practical value of this nomogram lies in its reliance on variables that are commonly available in routine clinical care. Unlike models requiring radiomics features, detailed volumetric measurements, or complete molecular profiles, this model can be applied using baseline clinical information and treatment records (20, 21). The web-based dynamic nomogram may further facilitate individualized risk estimation. In clinical practice, such a tool may help identify patients with poor expected survival who require closer supportive care and those with more favorable prognosis who may benefit from more active follow-up and systemic treatment planning.

Several limitations should be considered. First, this was a retrospective study from a single institution, and external validation in independent multicenter cohorts is needed before broader clinical application. Second, patients who did not complete the prescribed radiotherapy course were excluded, which may have reduced the proportion of patients with very poor performance status or rapidly progressive disease. Third, although radiotherapy modality was evaluated during variable selection and doses were converted to EQD2 where appropriate, the retrospective nature of the data limited a more detailed characterization of radiotherapy delivery. Broad categories such as WBRT, local radiotherapy, and WBRT combined with local boost may not fully capture differences in treatment technique, target coverage, dose distribution, fractionation, or biological effect. The potential influence of radiotherapy heterogeneity on survival should therefore be further examined in future studies with more detailed treatment-planning data. Fourth, detailed intracranial disease characteristics, including total tumor volume, neurological symptoms, and radiomic features, were not consistently available. Molecular information such as EGFR mutation, ALK rearrangement, and HER2 status was also incomplete and therefore could not be incorporated into the model. Finally, OS was defined as death from any cause, so intracranial progression-related deaths could not be distinguished from deaths mainly caused by extracranial disease progression. Because systemic therapies are rapidly evolving, particularly in lung and breast cancer, the prognostic impact of specific agents may also change over time and require future updates of the model.

## 5 Conclusion

This study developed a prognostic nomogram integrating six routinely available clinical and treatment-related variables to estimate OS in patients with BMs receiving radiotherapy. The model showed acceptable internal discrimination, calibration, and potential clinical usefulness in this single-center cohort. The nomogram-derived score may support individualized risk stratification and follow-up planning after radiotherapy. Further prospective multicenter validation is needed to evaluate its generalizability and refine its use in contemporary treatment settings.

## Data Availability

All original datasets that back up the findings presented in this manuscript can be provided freely by the corresponding author upon reasonable request.
